# Phthalate and Terephthalate Mixtures, Cardiometabolic Risk Factors, and the Role of Oxidative Stress: Evidence from NHANES 2015–2018

**DOI:** 10.1007/s12012-026-10188-7

**Published:** 2026-09-11

**Authors:** Mary D. Webb, Sheela Sathyanarayana, Drew B. Day, Jillian C. Trabulsi, Jee Won Park, Melissa M. Melough

**Affiliations:** 1 Department of Health Behavior and Nutrition Sciences, University of Delaware, Newark, DE 19713 USA; 2 Center for Child Health, Behavior and Development, Seattle Children’s Research Institute, Seattle, WA 98101 USA; 3 Department of Pediatrics, University of Washington, Seattle, WA 98195 USA; 4 Independent Researcher, Jersey City, NJ USA; 5 Department of Epidemiology, University of Delaware, Newark, DE 19713 USA

**Keywords:** Phthalates, EDCs, Glycemia, Lipids, Oxidative stress, Antioxidant balance

## Abstract

Phthalates are a class of endocrine-disrupting chemicals that may impair glucose and lipid metabolism through oxidative stress pathways. In this study, we analyzed NHANES 2015–2018 data on 2,336 U.S. adults and assessed fifteen phthalate and terephthalate metabolites, four glycemic outcomes, and five lipid outcomes. We used multivariable weighted quantile sum (WQS) regression to examine negative and positive associations of phthalate/terephthalate mixtures with glycemic and lipid outcomes. We assessed mediation by oxidative stress via gamma-glutamyltransferase (GGT) and explored modification by a composite score of dietary and lifestyle anti- and pro-oxidants (i.e., oxidative balance score, OBS) through the inclusion of product terms (WQS index*OBS) in linear regressions. A one-decile increase in phthalate/terephthalate mixtures dominated by mono(2-ethyl-5-hydroxyhexyl) terephthalate was associated (Estimate; 95% CI) with higher fasting glucose (1.95; 0.75, 3.14), fasting insulin (9.10%; 6.32%, 11.95%), HOMA-IR (9.86%; 6.86%, 12.94%), and HbA1c (0.05; 0.03, 0.07). Phthalate/terephthalate mixtures were associated with lower HDL-C (-0.93; -1.26, -0.60), LDL-C (-2.28; -3.54, -1.02), and total cholesterol (TC) (-1.89; -2.86, -0.93), and with higher fasting triglycerides (2.98%; 0.77%, 5.23%). GGT mediated a limited number of associations. OBS attenuated adverse associations between phthalate/terephthalate mixtures and select lipid outcomes, including HDL-C and TC/HDL-C ratio, among females. Overall, exposure to a mixture of phthalate/terephthalate metabolites was associated with disrupted glucose and lipid metabolism markers in this cross-sectional study. Oxidative stress may play a role in some of these associations, potentially providing a target for intervention, but longitudinal and randomized trials are needed to confirm these findings.

**Supplementary Information:**

The online version contains supplementary material available at https://doi.org/10.1007/s12012-026-10188-7.

## Introduction

 Cardiovascular disease (CVD) accounted for over 940,000 deaths in the United States (U.S.) in 2022 [1]. Prevention and management of CVD involves addressing major risk factors including hyperglycemia and dyslipidemia [1]. Certain environmental exposures, including to endocrine-disrupting chemicals (EDCs), are increasingly recognized for their adverse effects on these factors [2]. Phthalates are one class of EDCs that can dysregulate glucose and lipid metabolism [2], and in humans these chemicals have been associated with diabetes and insulin resistance [3–10] and with dyslipidemia [11–15]. Phthalates can induce oxidative stress which may contribute to altered glucose and lipid metabolism through impaired pancreatic *β*-cell function and insulin signaling [16, 17], diminished *Glut4* expression [16, 17], and damage to mitochondria and DNA [18].

Epidemiological studies also provide evidence that phthalate exposure may promote oxidative stress [7, 8, 10, 19–21], which may mediate associations between phthalate exposure and diabetes and insulin resistance [8, 10]. However, this body of evidence contains mixed findings, includes restricted samples from limited geographic locations and primarily assessed individual phthalate metabolites. Furthermore, existing studies assessing mediation have not included phthalate replacements/alternative plasticizers, an important limitation given temporal and geographical differences in industrial use of phthalates and their alternatives [22].

Importantly, there is also evidence that administration of antioxidants, including Pyrroloquinoline quinone (PQQ) [18] and vitamins C and E [23], can mitigate the oxidative stress-related effects of phthalates. Several nutrients (e.g., β-carotene, vitamins C and E) and physical activity have antioxidant properties [24, 25], while others (e.g., iron, smoking) act as pro-oxidants [26, 27]. Oxidative balance score (OBS), a composite score representing exposure to a combination of dietary and lifestyle antioxidants and pro-oxidants [28], has previously been associated with cardiometabolic improvements [29], yet the ability of OBS to attenuate the cardiometabolic effects of phthalate exposure has not been explored.

In the present study, we utilized the most recent NHANES data and a mixture exposure approach to examine the relationship of phthalate/terephthalate mixtures with cardiometabolic risk factors and to explore the mediating role of oxidative stress and the modifying role of OBS. We hypothesized that exposure to a mixture of phthalates/terephthalates would be associated with adverse glycemic and lipid profiles and that oxidative stress (assessed using gamma-glutamyltransferase (GGT)) would mediate these effects while greater antioxidant exposure (assessed using OBS) would attenuate the adverse associations.

## Methods

### Participants

NHANES is a national survey of the noninstitutionalized U.S. civilian population [30]. The present analyses included male and nonpregnant female adults (aged ≥ 20 years) from NHANES 2015–2018. Eligible participants had complete data available on all included phthalate and terephthalate metabolites, data on at least one outcome, and complete data on covariates. After applying these criteria, 2,336 participants were eligible for at least one of the glycemic (*N* = 2,334) or lipid analyses (*N* = 2,335) in Aim 1 (Fig. S1). For Aim 2 that examined mediation by oxidative stress, a total of 2,224 participants were eligible after excluding those with a liver condition. All participants eligible for Aim 1 were also eligible for Aim 3 exploring effect modification by OBS (Fig.S1). NHANES protocols were approved by the National Center for Health Statistics Ethics Review Board, and all participants provided informed consent. The present secondary analysis of de-identified NHANES data was conducted in accordance with the Declaration of Helsinki.

### Urinary Phthalate and Terephthalate Metabolites

Urinary phthalate and terephthalate metabolites were measured in spot urine samples and analyzed at the National Center for Environmental Health, as previously described [31]. Phthalate and phthalate replacement metabolites with at least 70% detection were included, and concentrations below the lower limit of detection (LLOD) were imputed by NHANES as the LLOD divided by the square root of 2 [32]. A total of 15 metabolites were included in the present analyses, including 13 phthalate and 2 terephthalate metabolites, as described in Table 1.

To account for urine dilution, we used covariate-adjusted standardization [33]. Covariate-adjusted creatinine-standardized phthalate metabolite concentrations (*E*_*Cr*_) were calculated according to following formula [33]: $\:{E}_{Cr}=\:{E}_{o}\times\:{(Cr}_{p}/{Cr}_{o})$, where *E*_*o*_ represents the observed phthalate/terephthalate metabolite concentration, *Cr*_*p*_ is the predicted creatinine concentration, and *Cr*_*o*_ is the observed urinary creatinine concentration. To generate *Cr*_*p*_ values, urinary creatinine was regressed on age, sex, race and ethnicity, body mass index (BMI), height, time of day of examination session, and estimated glomerular filtration rate [34]. Additionally, we adjusted for natural log-transformed urinary creatinine in all analyses [33].

### Outcomes

#### Glycemic Outcomes

We assessed four glycemic outcomes: fasting plasma glucose (mg/dL), fasting insulin (µU/mL), homeostatic model assessment of insulin resistance (HOMA-IR), and glycated hemoglobin (HbA1c, %). Fasting plasma glucose, fasting insulin, and HbA1c were measured from blood specimens. HOMA-IR was calculated as the product of fasting insulin (µU/mL) and fasting glucose (mmol/L), divided by 22.5 [35]. For the glucose, insulin, and HOMA-IR outcomes, participants were required to have fasted between 8 and 24 h.

#### Lipid Outcomes

The five lipid outcomes assessed included high-density lipoprotein cholesterol (HDL-C) (mg/dL), low-density lipoprotein cholesterol (LDL-C) (mg/dL), total cholesterol (TC) (mg/dL), TC to HDL-C (TC/HDL-C) ratio, and fasting triglycerides (TG) (mg/dL). Serum LDL-C was calculated by NHANES from TC, HDL-C, and TG values using the Friedewald Equation [36]. Fasting requirements were applied to the TG and LDL-C outcomes.

### Serum GGT

To investigate how oxidative stress may contribute to the associations of phthalate exposure with glycemic and lipid outcomes, serum gamma-glutamyltransferase (GGT) (U/L) was considered as a mediator. GGT is an enzyme involved in glutathione metabolism and has been proposed as a biomarker of oxidative stress, as higher GGT concentrations within the normal range have been associated with increased oxidative damage [37].

### Oxidative Balance Score (OBS)

OBS was calculated using 16 antioxidant and 5 pro-oxidant dietary and lifestyle components (Table S1), adapted from Zhang et al. [38]. Dietary antioxidants, assessed from 24-hour recalls, included β-carotene, calcium, copper, fiber, folate, magnesium, niacin, EPA + DHA, riboflavin, selenium, Vitamin B_12_, Vitamin B_6_, Vitamin C, Vitamin E, and zinc, while the dietary pro-oxidants were iron and saturated fat. Lifestyle components included one antioxidant factor (physical activity) and three pro-oxidant factors (alcohol consumption, smoking status, BMI). Sex-specific tertiles were created for all components except alcohol consumption and smoking status, which were scored according to preestablished criteria (Table S1). Antioxidant components were scored in ascending order (0, 1, 2 points) while descending scoring was applied to most pro-oxidants (2, 1, 0 points). The total OBS was determined as the sum of the points from the 21 contributing components.

### Covariates

Covariates were selected based on the existing literature and included sex, age, race and ethnicity, education level, family income to poverty ratio (PIR), smoking status, alcohol consumption, physical activity, sedentary behavior, total daily energy intake, diet quality, natural log-transformed urinary creatinine, menopause status, survey cycle, and glucose- or lipid-lowering medication use, for the glucose and lipid outcomes, respectively.

### Statistical Analyses

All analyses were conducted with R (v4.5.1; R Development Core Team 2025). We calculated descriptive statistics to summarize the distributions of phthalate/terephthalate exposures, outcomes, and covariates in the study population.

To examine associations between mixtures of phthalate and terephthalate metabolites with cardiometabolic risk factors and the relative contribution of each metabolite to the mixture, we used multivariable weighted quantile sum (WQS) regressions (WQSr) [39]. These were run without splitting the data into training and validation data sets to maximize statistical power, and the valid p-values were derived for WQSr coefficients using a permutation test as implemented in the *wqspt* R package [40]. We performed these permutation tests with 200 iterations when the naïve 95% CIs of the WQSr models did not include the null. We performed WQSr in the positive and negative directions given prior inconsistencies in the direction of associations between phthalate exposure and glycemic and lipid outcomes. In the positive-direction model, the WQS index was constrained to identify a mixture associated with higher outcome values, whereas in the negative-direction model, the WQS index was constrained to identify a mixture associated with lower outcome values. Consequently, the relative metabolite weights can differ between the models. WQSr models were also run stratified by sex. Natural log-transformed phthalate metabolite concentrations were decile-transformed for WQSr.

As a supplemental analysis, we fit multivariable linear regression models for each individual phthalate and terephthalate metabolite in relation to each glycemic and lipid outcome. These models were adjusted for the same covariates as the corresponding WQS models and were used to qualitatively evaluate the consistency of the directions of association across individual metabolites.

To assess whether the associations between phthalate/terephthalate mixtures and cardiometabolic risk factors operate through serum GGT (a proxy of oxidative stress), we estimated the average causal mediation effect (ACME), average direct effect (ADE), and total effect using the *mediation* R package [41] and bootstrapping with 1,000 iterations. The percent mediated was also calculated [$\:(ACME/(Total\:Effect\left)\right)*100]$. For mediation analyses, we excluded participants who reported having a liver condition.

We also included product terms between the WQSr-derived WQS index and OBS (WQS index*OBS) in multivariable linear regression models to explore effect modification. Physical activity, alcohol consumption, and smoking status were removed as covariates in these models as they are components of OBS. When the product term p-value was < 0.05, we also generated plots of the WQS index across levels of OBS.

### Sensitivity Analyses

To assess the robustness of our findings, we repeated the WQS analyses with the inclusion of BMI and multiple medical conditions: self-reported liver condition, kidney dysfunction, diabetes, and hypertension. Self-reported CVD was also included in the sensitivity analyses for the lipid outcomes. We performed the sensitivity analyses by adding BMI, the medical conditions, or both as covariates. These covariates were limited to sensitivity analyses because they may lie on the causal pathway between phthalate exposure and the assessed outcomes. A lack of substantial changes in coefficient directions or effect sizes after performing these sensitivity analyses would be interpreted as evidence that the added covariates are neither confounders nor mediators.

We conducted additional mediation analyses with generalized additive models (GAMs) based on prior evidence of nonlinear associations between phthalate exposure and GGT [42, 43]. GAMs were used for the mediator and outcome models and the same covariates used in the linear mediation models were applied to these models.

We also calculated two additional OBS: dietary OBS (DOBS) and OBS plus dietary supplements (OBS+) to examine whether the modification was impacted by inclusion of only dietary components or with the addition of dietary supplements. DOBS was calculated based on the 17 dietary antioxidants and pro-oxidants described in Sect.  2.5. For OBS+, the dietary components were based on combined dietary and supplemental nutrient intake, while the lifestyle components remained the same.

## Results

### Characteristics of the Study Participants

Among the 2,336 eligible participants, (Fig. S1), 49.2% were female and the average age was 47.9 ± 17.0 years. Participants were primarily non-Hispanic White (65.4%) and educated at an above high school level (64.7%) (Table 2). Glycemic and lipid levels tended to fall outside of values consistent with diabetes/insulin resistance and dyslipidemia. Median (25th, 75th percentile) serum GGT levels (U/L) were 20.0 (14.0, 31.9) and OBS values ranged from 5 to 39, with a mean of 22.1 ± 7.3 (Table 2).

### Phthalate and Terephthalate Exposure

Of the 15 metabolites included, the detection rate ranged from 70.8% (MHBP) to 99.9% (MECPTP) (Table 1). The covariate-adjusted creatinine-standardized urinary metabolite concentrations were lowest for MHBP (geometric mean (GM): 0.8 ng/mL) and highest for MEP (GM: 31.5 ng/mL) (Table 1).

### Associations Between Phthalates/Terephthalates and Cardiometabolic Risk Factors

#### Glycemic Outcomes

**Fig. 1:**
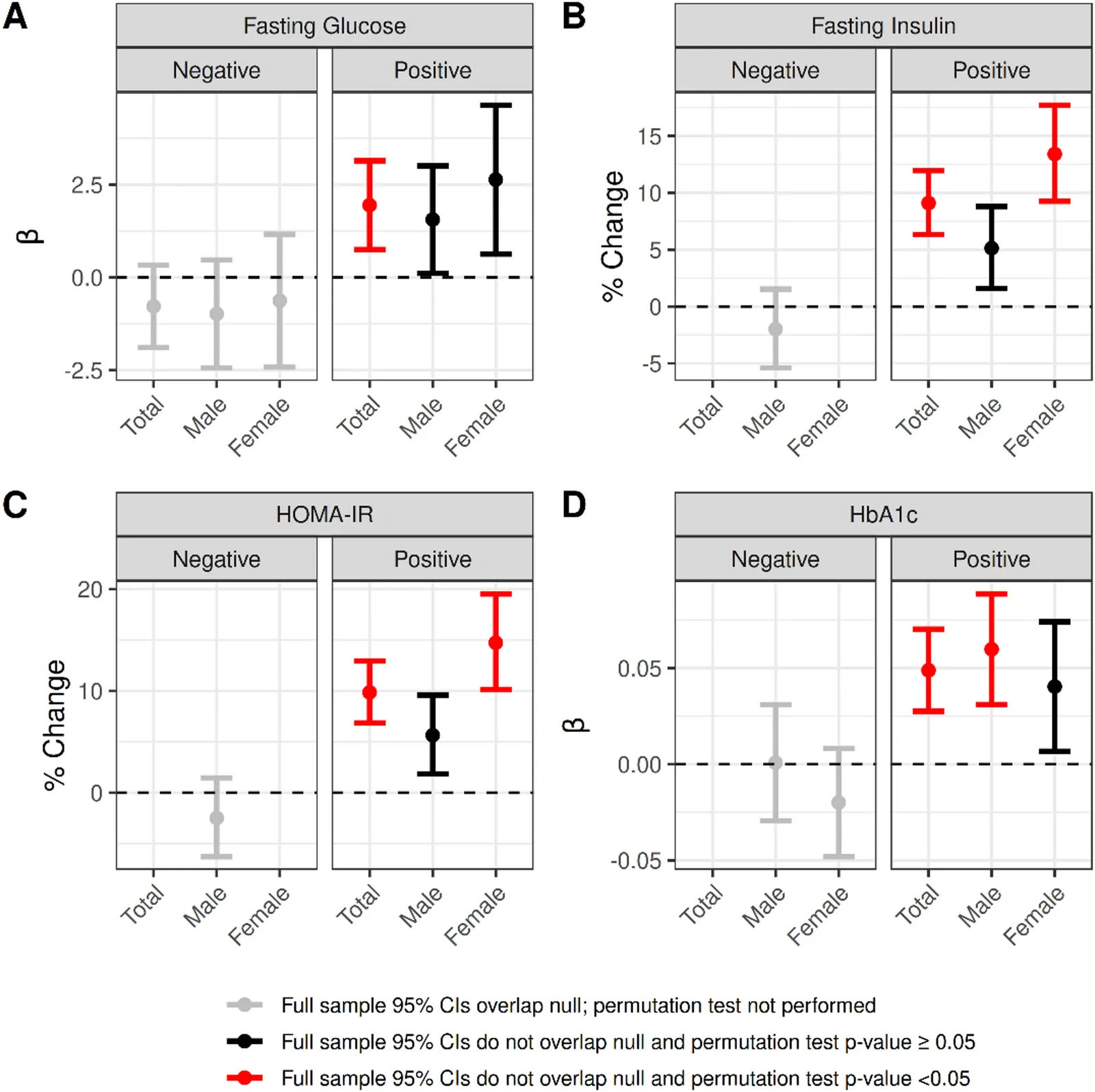
Effect estimates (and 95% CIs) from WQS regression models for associations between phthalate and terephthalate mixtures and **A** fasting glucose (*N* = 1,090 ), **B** fasting insulin (*N* = 1,086), **C** HOMA-IR (*N* = 1,085), and **D** HbA1c (*N* = 2,333) among all eligible NHANES 2015–2018 participants and stratified by sex. Models were adjusted for sex (total models with both sexes), age, race and ethnicity, education level, PIR, smoking status, alcohol consumption, physical activity, sedentary behavior, energy intake, diet quality, natural log-transformed urinary creatinine, menopause status, survey cycle, and glucose-lowering medication use. Plot columns indicate the outcome assessed and the direction of the WQS regression models and the three results within each of these columns refer to models run with both sexes combined or stratified by sex. Effect estimates represent the unit or percent change (fasting insulin, HOMA-IR) in the outcome for a one-decile increase in the phthalate/terephthalate mixture. Blank spaces indicate models for which the directionally constrained WQS regression did not produce a valid estimate and therefore no effect estimate is presented

In the models examining all participants, phthalate and terephthalate mixtures demonstrated significant positive associations with glycemic outcomes. Namely, a one-decile increase in the phthalate and terephthalate mixture was associated with 1.95 mg/dL higher fasting glucose, (95% CI: 0.75, 3.14; permutation test p-value (PTp) = 0.03), 9.10% higher fasting insulin (95% CI: 6.32%, 11.95%; PTp < 0.005), 9.86% higher in HOMA-IR (95% CI: 6.86%, 12.94%; PTp < 0.005), and 0.05 higher HbA1c (95% CI: 0.03, 0.07; PTp = 0.005) (Fig. 1A-D). The highest weights were observed for MEHHTP, while some low molecular weight (LMW) metabolites (MEP for glucose, MiBP for insulin, HOMA-IR, and HbA1c), and some DEHP metabolites (MECPP for glucose, MEHHP for insulin and HOMA-IR, MEOHP for HbA1c) were also highly weighted (Fig. S2). In sex-stratified models, overall associations largely remained the same, but the precision of the WQSr results were less supported by permutation tests. Associations with fasting glucose were no longer supported by permutation tests among either sex (Fig. 1A), while associations remained statistically significant after the permutation test among only females for fasting insulin (β_females_ = 13.41%, 95% CI: 9.26%, 17.71%; PTp < 0.005) and HOMA-IR (β_females_ = 14.73%, 95% CI: 10.13%, 19.52%; PTp < 0.005) (Fig. 1B-C). For HbA1c, findings remained significant only among males (β_males_ = 0.06, 95% CI: 0.03, 0.09; PTp < 0.005) (Fig. 1D). No significant inverse associations were observed between mixture exposures and the glycemic outcomes (Fig. 1A-D).

#### Lipid Outcomes

**Fig. 2:**
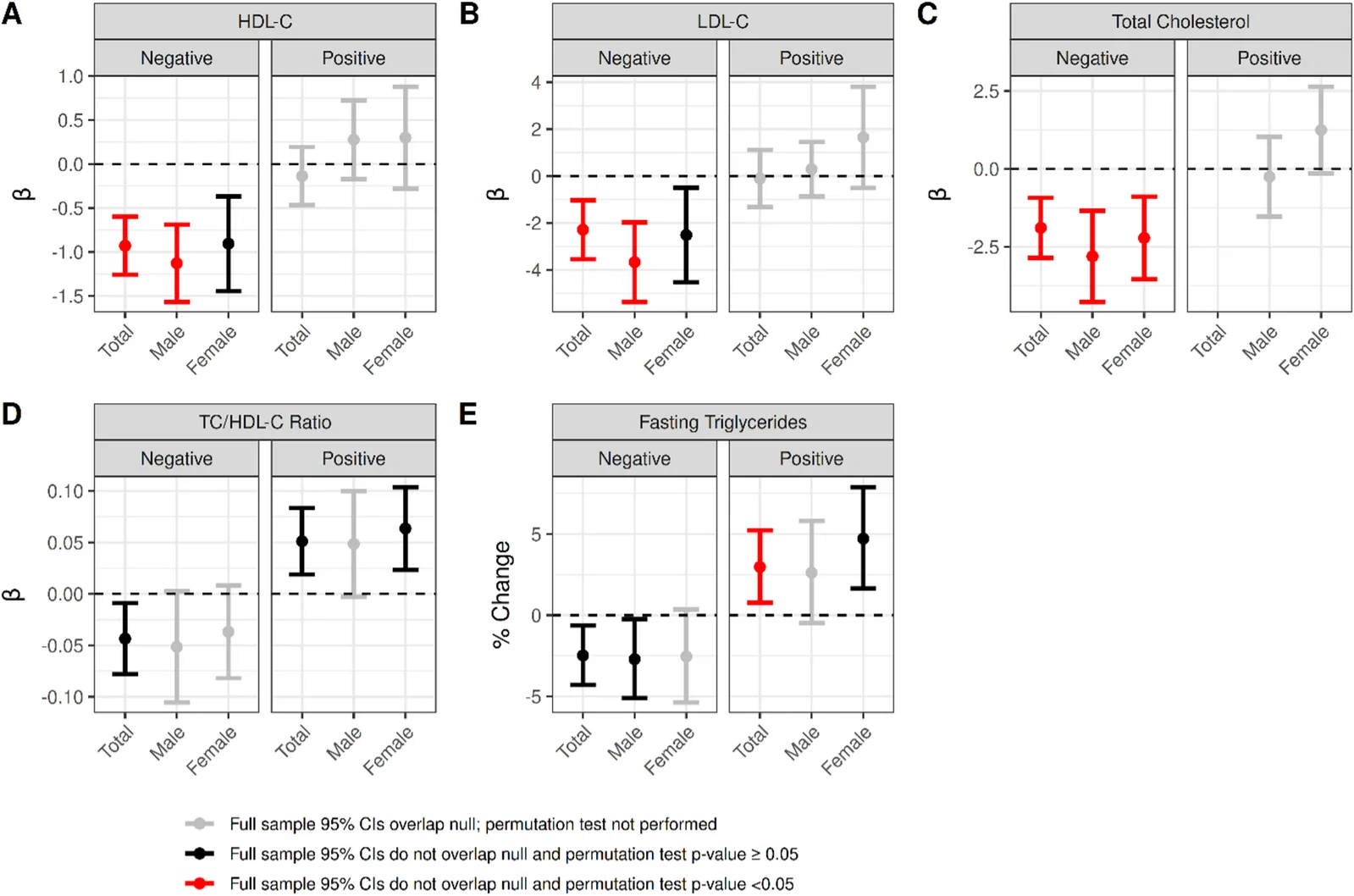
Effect estimates (and 95% CIs) from WQS regression models for associations between phthalate and terephthalate mixtures and **A** HDL-C (*N* = 2,335), **B** LDL-C (*N* = 1,073), **C** total cholesterol (*N* = 2,335), **D** total cholesterol to HDL-C ratio (*N* = 2,335), and **E** fasting triglycerides (*N* = 1,090) among all eligible NHANES 2015–2018 participants and stratified by sex. Models were adjusted for sex (total models with both sexes), age, race and ethnicity, education level, PIR, smoking status, alcohol consumption, physical activity, sedentary behavior, energy intake, diet quality, natural log-transformed urinary creatinine, menopause status, survey cycle, and lipid-lowering medication use. Plot columns indicate the outcome assessed and the direction of the WQS regression models and the three results within each of these columns refer to models run with both sexes combined or stratified by sex. Effect estimates represent the unit or percent change (fasting triglycerides) in the outcome for a one-decile increase in the phthalate/terephthalate mixture. Blank spaces indicate models for which the directionally constrained WQS regression did not produce a valid estimate and therefore no effect estimate is presented

A one-decile increase in the exposure mixture was associated with 2.98% higher fasting TG among all participants (95% CI: 0.77%, 5.23%; PTp = 0.035) (Fig. 2E) and the highest weights were for MBzP, MEHHTP, and MCiOP (Fig. S3). No additional positive associations among the total sample were supported as significant by permutation tests. When assessing the effects in the negative direction among all participants, we found that a one-decile increase in the phthalate and terephthalate mixture was associated with 0.93 mg/dL lower HDL-C (95% CI: -1.26, -0.60; PTp < 0.005), 2.28 mg/dL lower LDL-C (95% CI: -3.54, -1.02; PTp = 0.02), and 1.89 mg/dL lower TC (95% CI: -2.86, -0.93; PTp = 0.01) (Fig. 2A-C). Among these significant inverse associations in the total sample, the metabolites with the highest weight among the mixture differed by outcome (HDL-C: MCiOP, MECPP, MBzP; LDL-C: MECPTP, MEHHTP, MCPP; TC: MOiNP, MHiBP, MEOHP) (Fig. S3). When stratifying by sex, inverse associations remained significant among males for HDL-C (β_males_ = -1.13, 95% CI: -1.57, -0.69; PTp < 0.005) and LDL-C (β_males_ = -3.66, 95% CI: -5.35, -1.97; PTp < 0.005) (Fig. 2A-B) and among both sexes for TC (β_males_ = -2.81, 95% CI: -4.27, -1.35; PTp = 0.005; β_females_ = -2.22, 95% CI: -3.55, -0.90; PTp = 0.045) (Fig. 2C).

Sensitivity analyses generally confirmed the robustness of our findings. When including BMI and/or medical conditions as covariates, results were largely consistent with our primary analyses (Fig. S4 & S5). However, the magnitude of the coefficients tended to be attenuated with the inclusion of BMI, particularly for the glycemic outcomes. Individual metabolite analyses (Fig. S6) demonstrated generally consistent directions of association across metabolites, supporting the directional homogeneity assumption underlying the WQS models.

### Mediation by Serum GGT

**Fig. 3:**
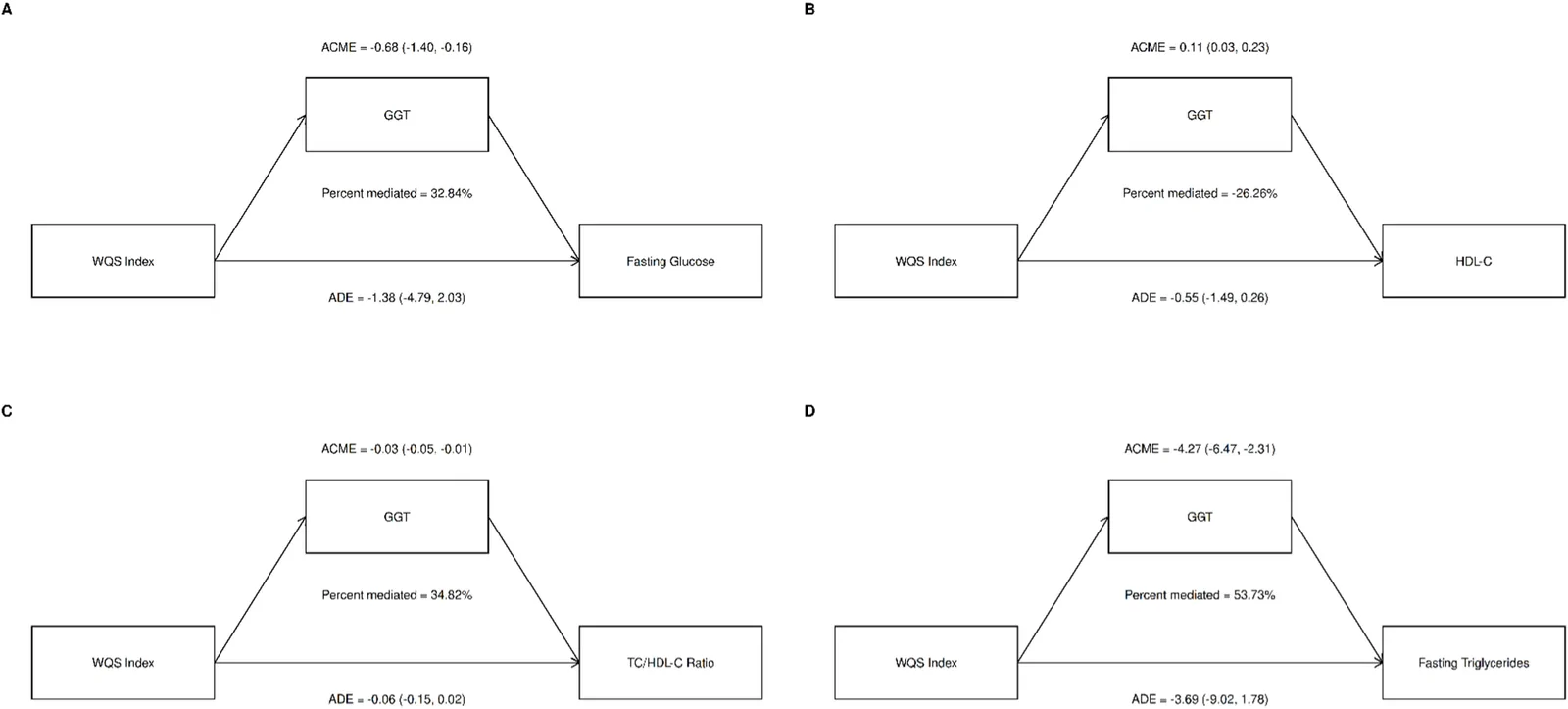
Mediation analysis of gamma-glutamyltransferase (GGT) in the associations between phthalate and terephthalate mixtures and **A** fasting glucose (negative direction) (*N* = 1,034 ), **B** HDL-C (positive direction) (*N* = 2,223), **C** total cholesterol to HDL-C ratio (negative direction) (*N* = 2,223), and **D** fasting triglycerides (negative direction) (*N* = 1,034). Models were adjusted for sex, age, race and ethnicity, education level, PIR, smoking status, alcohol consumption, physical activity, sedentary behavior, energy intake, diet quality, natural log-transformed urinary creatinine, menopause status, survey cycle, and glucose-lowering (glycemic outcomes) or lipid-lowering (lipid outcomes) medication use. Mediation analyses were conducted using 1,000 bootstrap samples to estimate the average direct effect (ADE), average causal mediation effect (ACME), and total effect. Mediation effect estimates presented represent the unit or percent (fasting triglycerides) change in the outcome for a change from the first to third quartile of the phthalate/terephthalate mixture exposure (i.e., WQS index). The percent mediated ((ACME /total effect) * 100) is also reported

In the mediation analyses, GGT was identified as a statistically significant mediator of the associations between phthalate/terephthalate exposures and fasting glucose (negative direction), HDL-C (positive direction), TC/HDL-C ratio (negative direction), and fasting TG (negative direction) (Fig. 3 & Table S2). For example, for a change from the first (3.0) to third quartile (6.0) of the negative-direction WQS index, fasting TG are estimated to be 4.27% lower through the exposure mixture’s association with GGT, assuming all other variables are constant, while fasting TG are estimated to be 3.69% lower through the direct association with the exposure mixture, independent of GGT (Fig. 3D). Overall, GGT accounted for 34.82% and 53.73% of the associations between the phthalate/terephthalate mixtures and lower TC/HDL-C ratio (Fig. 3C) and fasting TG (Fig. 3D), respectively. Our findings were similar when assessing mediation with GAMs and we did not observe considerable evidence of non-linear associations.

### Modification by OBS

**Fig. 4:**
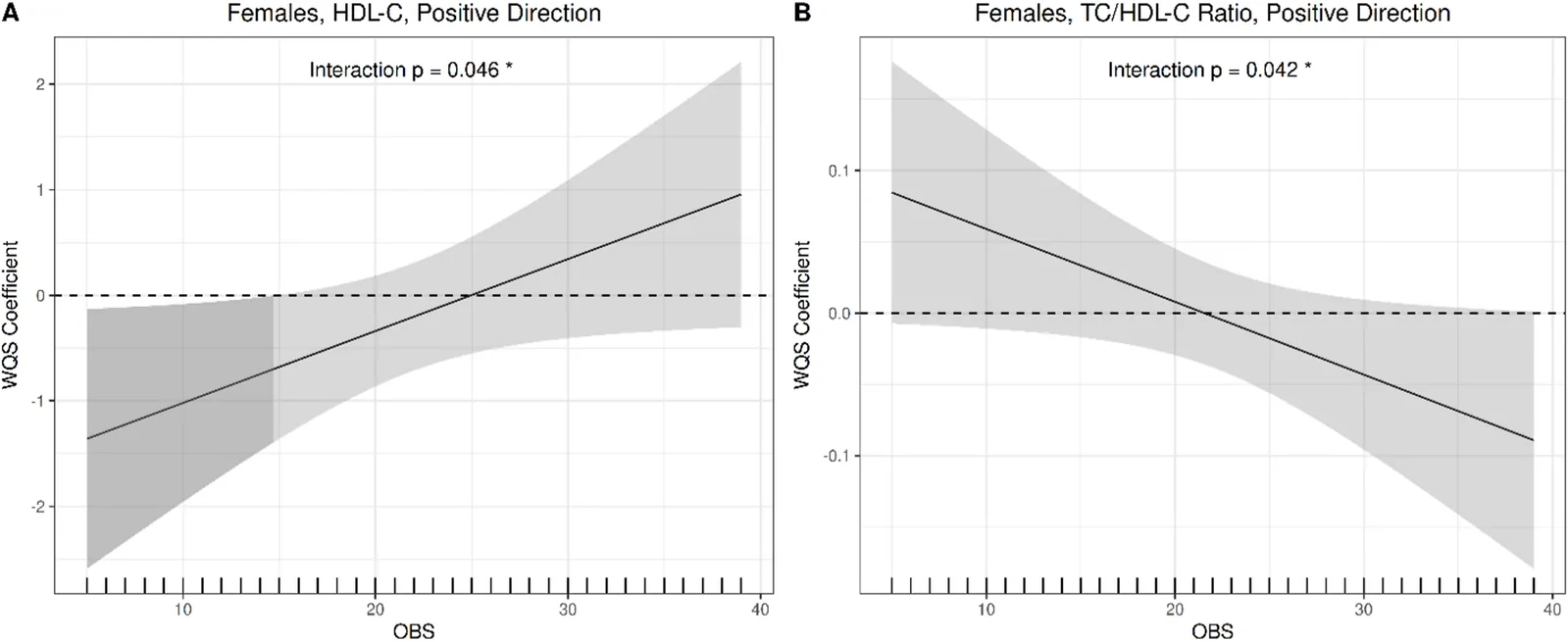
Predicted phthalate and terephthalate WQS coefficients and 95% CIs in association with **A** HDL-C (*N* = 1,150) and **B** total cholesterol to HDL-C ratio (*N* = 1,150) among females in the positive direction over values of oxidative balance score (OBS) in interaction models. Models were adjusted for age, race and ethnicity, education level, PIR, sedentary behavior, energy intake, diet quality, natural log-transformed urinary creatinine, menopause status, survey cycle, and lipid-lowering medication use. All models also included a product term between the WQS mixture coefficient and OBS. Plots are shown for models in which the product term was statistically significant (*p* < 0.05). P-values for the product term (interaction p) are shown within each plot

Among all participants, we did not observe significant effect modification by OBS. However, in sex-stratified models, OBS significantly modified the associations between the phthalate/terephthalate mixture and HDL-C and TC/HDL-C ratio among females (Fig. 4). We observed that the association between the exposure mixture and HDL-C was negative at lower OBS but this association was attenuated at higher scores (Fig. 4A). For TC/HDL-C ratio, as the OBS increased, the association between the exposure mixture and TC/HDL-C ratio became more negative (Fig. 4B).

We also assessed effect modification by an OBS with only dietary components (DOBS) or an OBS that also considered dietary supplement intake (OBS+). Beyond those outcomes also modified by OBS, DOBS and OBS+ modified associations with fasting TG among males (Fig. S7D) and TC among females (Fig. S7F), respectively, in the same pattern described above for OBS with TC/HDL-C ratio.

**Table 1:** Phthalate and phthalate replacement metabolite concentrations (ng/mL)^a^

Phthalate and phthalate replacement metabolites	Detection frequency, %	GM	25^th^ percentile	Median	75^th^ percentile
Monoethyl phthalate (MEP)	99.6	31.5	13.0	26.9	61.8
Mono-n-butyl phthalate (MBP)	99.2	8.7	5.7	8.7	13.2
Mono-3-hydroxybutyl phthalate (MHBP)	70.8	0.8	0.5	0.8	1.2
Monoisobutyl phthalate (MiBP)	97.4	7.3	4.4	7.0	11.7
Mono-2-hydroxy-isobutyl phthalate (MHiBP)	93.9	2.3	1.3	2.2	3.5
Monobenzyl phthalate (MBzP)	96.0	3.3	1.6	3.0	6.5
Mono(2-ethyl-5-oxohexyl) phthalate (MEOHP)	99.2	3.0	1.9	2.9	4.7
Mono(2-ethyl-5-hydroxyhexyl) phthalate (MEHHP)	99.0	4.9	3.0	4.7	7.4
Mono(2-ethyl-5-carboxypentyl) phthalate (MECPP)	99.8	7.5	4.7	7.1	11.0
Mono(2-ethyl-5-hydroxyhexyl) terephthalate (MEHHTP)	93.9	5.6	2.1	4.6	12.7
Mono(2-ethyl-5-carboxypentyl) terephthalate (MECPTP)	99.9	18.9	6.6	16.0	50.6
Mono(3-carboxypropyl) phthalate (MCPP)	78.7	1.1	0.6	1.0	1.7
Monooxoisononyl phthalate (MOiNP)	87.8	1.6	0.8	1.4	2.4
Monocarboxyisooctyl phthalate (MCiOP)	99.3	6.2	3.0	4.9	10.0
Monocarboxyisononyl phthalate (MCiNP)	96.3	1.5	0.8	1.4	2.4
Mono(2-ethylhexyl) phthalate (MEHP)*	55.8	1.2	0.6	1.1	2.0
Monoisononyl phthalate (MiNP)*	18.4	0.9	0.5	0.8	1.4
Cyclohexane-1,2-dicarboxylic acid monohydroxyisononyl ester (MHiNCH)*	56.6	0.7	0.3	0.6	1.2
Cyclohexane-1,2-dicarboxylic acid monocarboxyisooctyl ester (MCOCH)*	41.9	0.6	0.3	0.6	1.1

^a^Covariate-adjusted creatinine-standardized concentrations (O’Brien et al., 2016).

*Excluded from analyses due to <70% of measurements at or above detection limit.

*GM* Geometric mean.

**Table 2:** Characteristics of the eligible study participants from NHANES 2015–2018 (*N* = 2,336)

Characteristic	Mean (SD)	Median (25th, 75th)
Age (years)	47.9 (17.0)	48.0 (33.0, 61.0)
Sedentary behavior (min/day)	367.1 (199.8)	360.0 (240.0, 480.0)
Energy intake (kcal/day)	2,121.5 (821.9)	2,001.0 (1,557.5, 2,562.5)
HEI-2015 total score (0–100)	52.9 (13.9)	51.6 (42.7, 62.8)
Urinary creatinine (mg/dL)	123.1 (81.0)	108.0 (60.0, 168.0)
Fasting glucose (mg/dL) (*N* = 1,090)	110.5 (33.2)	103.0 (95.0, 112.0)
Fasting insulin (µU/mL) (*N* = 1,086)	13.3 (16.9)	9.3 (5.8, 16.0)
HOMA-IR (*N* = 1,085)	4.0 (6.8)	2.4 (1.4, 4.4)
HbA1c (%) (*N* = 2,333)	5.6 (0.9)	5.5 (5.2, 5.8)
HDL-C (mg/dL) (*N* = 2,335)	54.5 (17.4)	51.0 (42.0, 63.0)
LDL-C (mg/dL) (*N* = 1,073)	111.2 (35.6)	107.0 (85.0, 130.0)
Total cholesterol (mg/dL) (*N* = 2,335)	191.2 (42.5)	187.0 (162.0, 216.0)
Total cholesterol/HDL-C ratio (*N* = 2,335)	3.8 (1.6)	3.5 (2.8, 4.6)
Fasting triglycerides (mg/dL) (*N* = 1,090)	112.4 (83.0)	90.0 (59.0, 142.0)
Gamma-glutamyltransferase (GGT) (U/L)	29.7 (31.8)	20.0 (14.0, 31.9)
Oxidative balance score (0–42)	22.1 (7.3)	23.0 (16.0, 28.0)
*Characteristic*	*N (%)*	
*Survey Cycle*		
2015/2016	1,121 (51.3%)	
2017/2018	1,215 (48.7%)	
*Sex*		
Male	1,186 (50.8%)	
Female	1,150 (49.2%)	
*Race and ethnicity*		
Non-Hispanic Asian	245 (4.7%)	
Hispanic	607 (14.7%)	
Non-Hispanic White	867 (65.4%)	
Non-Hispanic Black	508 (10.6%)	
Other	109 (4.7%)	
*Education level*		
< HS	452 (10.9%)	
HS or equivalent	539 (24.3%)	
> HS	1,345 (64.7%)	
*Family income to poverty ratio*		
≤ 1.30	699 (19.6%)	
> 1.30 to ≤ 3.50	923 (34.3%)	
> 3.50	714 (46.1%)	
*Physical activity level* (MET-min/week)		
< 500	825 (30.5%)	
500–1000	223 (9.3%)	
> 1000	1,288 (60.2%)	
*Smoking status*		
Never	1,315 (58.2%)	
Former	573 (24.0%)	
Current	448 (17.7%)	
*Alcohol consumption*		
None/never	776 (24.9%)	
Moderate	790 (37.8%)	
Above moderate	770 (37.3%)	
*Menopause status*		
Premenopausal/NA (males)	1,725 (74.6%)	
Postmenopausal	611 (25.4%)	
*Glucose-lowering medication use*		
Not currently using	2,030 (90.9%)	
Currently using	306 (9.1%)	
*Lipid-lowering medication use*		
Not currently using	1,863 (83.2%)	
Currently using	473 (16.8%)	

Data are presented as weighted mean (SD) and weighted median (25th, 75th percentile) for continuous variables and as unweighted counts (weighted percentages) for categorical variables

*HOMA-IR* Homeostatic Model Assessment for Insulin Resistance, *HbA1c* glycated hemoglobin, *HDL-C* high-density lipoprotein cholesterol, *LDL-C* low-density lipoprotein cholesterol, *MET* metabolic equivalent of task

## Discussion

In the present analyses, we observed significant associations between phthalate/terephthalate mixtures dominated by the terephthalate metabolite MEHHTP and higher glycemic outcomes. We also discovered numerous significant associations with lipid outcomes as the phthalate/terephthalate mixtures demonstrated inverse associations with HDL-C, LDL-C and TC, and positive associations with fasting TG. Serum GGT, used as a proxy of oxidative stress, was identified as a statistically significant mediator in the associations between the phthalate/terephthalate mixture exposure and select glycemic and lipid outcomes. We found that OBS, a comprehensive score indicative of greater antioxidant exposure, attenuated the unfavorable associations of phthalate/terephthalate mixtures with HDL-C and TC/HDL-C ratio among females.

We observed consistent positive associations between exposure to phthalate/terephthalate mixtures and glycemic outcomes. Similar findings have been reported in some studies across a variety of populations including adolescents [3, 5, 6], adults [4–6, 10], and the elderly [7]. Phthalate exposure may alter glucose and insulin-related biomarkers by interfering with various pathways including sex steroid [44], thyroid [45], peroxisome proliferator-activated receptor (PPAR) [46], and oxidative stress pathways [7, 8, 18, 23].

The mixtures associated with glucose outcomes in the present analyses were consistently dominated by MEHHTP, a metabolite of di(2-ethylhexyl) terephthalate which belongs to a class of alternative plasticizers called terephthalates. Terephthalate exposure has previously been linked with impaired glycemic outcomes in women during pregnancy [47]. Terephthalates may act through similar mechanisms to phthalates, including through disruptions to sex steroid hormones [48, 49] and oxidative stress and inflammation [21, 43].

We also observed notable contributions of MEP to the association with fasting glucose and MiBP with fasting insulin, HOMA-IR, and HbA1c. MEP exposure has previously been implicated in impaired glycemic control among pregnant women [50], and measures of diabetes and insulin resistance or signaling among adolescents [5, 6] and adults [4–6]. Furthermore, in vitro work has demonstrated the ability of MEP to affect insulin secretion from pancreatic β-cells [51]. Experimental studies have identified estrogenic activity [51], PPAR agonism [51], and oxidative stress [52] as potential links between MEP (or its parent compound) exposure and impaired insulin signaling. Interestingly, among mixtures associated with fasting insulin, HOMA-IR, and HbA1c in the present analyses, MEP was not a notable contributor. Instead, MiBP, another LMW phthalate metabolite, was implicated in these mixtures. This finding was consistent with previous epidemiological studies linking MiBP exposure with elevated insulin [6], insulin resistance [3, 6], and HbA1c [5, 6]. Animal and in vitro evidence has demonstrated that LMW phthalate metabolites (e.g., MEP, MBP, and MiBP) preferentially bind to PPARα [46, 53] which may also contribute to the undesirable effects observed for glucose and insulin markers [10].

The various associations we observed between phthalate mixture exposures and lipid outcomes may be due in part to the effects of PPARs. Phthalates promote adipogenesis and lipid accumulation in part through their activation of PPARs including PPARγ, a main regulator of adipocyte differentiation [46, 54]. Some phthalates monoesters may also activate PPARα [46, 54] which can contribute to fatty acid oxidation and decreased lipid levels [55]. This activation of PPARα may further contribute to some of the lower LDL-C and TC levels we observed in association with an increase in exposure to a mixture of phthalate metabolites. In addition to the effects of PPARs, another possible biological explanation comes from animal data showing that DEHP inhibits hepatic cholesterol biosynthesis, potentially through inhibition of the rate-limiting enzyme HMG-CoA reductase [56], potentially through inhibition of the rate-limiting enzyme HMG-CoA reductase, which could contribute to lower circulating concentrations of TC and LDL-C. Prior epidemiological analyses have demonstrated associations with lower HDL-C [13, 15], LDL-C [11, 14], and TC [14], and higher TG [12, 15]. However, some studies have reported positive associations for HDL-C [11, 14] and TC [12] and inverse associations for TG [11], while others report overall null findings for lipid outcomes [57]. These discrepant findings may be related to the heterogeneity among the study populations, timing of exposure assessment relative to outcome assessment, and methods of exposure assessment and analysis. Our findings add to the existing body of literature that exposure to a mixture of phthalates and terephthalates can contribute to dysregulated lipid metabolism.

Our mediation analyses indicated that serum GGT significantly mediated the association between phthalate/terephthalate mixtures and a few cardiometabolic outcomes. GGT is critical to extracellular GSH metabolism and regeneration of intracellular GSH, a key antioxidant, and products of extracellular GSH metabolism can produce ROS, especially in the presence of transition metals like iron [37]. Some individual phthalate metabolites have shown positive associations with GGT [42, 43, 58], while others negative [42, 43]. We observed that serum GGT significantly mediated negative phthalate mixture associations with fasting TG. Phthalates and terephthalates may exert hypolipidemic effects through their ability to activate PPARs [46, 54] whose activation can lead to lower serum FFA and TG levels in liver and muscle, but increased adipose tissue mass [59]. Among prior studies, different metabolites have had varied associations with GGT [42, 43], and associations were null for mixtures among those that utilized mixture analyses [43, 58]. Therefore, the incorporation of multiple metabolites into a mixture that may exert varying effects on GGT may have contributed to null findings in our analyses.

Through our modification analyses, we found that OBS significantly modified the associations between phthalate/terephthalate mixture exposure and HDL-C and TC/HDL-C ratio among females. At higher OBS levels, indicating greater antioxidant exposure [28], there were improved levels of HDL-C (i.e., higher) and TC/HDL-C ratio (i.e., lower). OBS has previously been shown protective against low HDL-C in some [60, 61] but not all [62] prior analyses. Components of the OBS used in the present analyses include dietary antioxidants and physical activity which can increase HDL-C levels [63, 64]. Thus, higher OBS, reflecting greater antioxidant exposure, likely contributed to a relative improvement of TC/HDL-C ratio by attenuating the decrease in HDL-C observed with phthalate/terephthalate exposure in the main analyses. OBS did not modify associations between phthalate/terephthalate mixture exposure and any additional cardiometabolic risk factors. Past studies exploring antioxidant nutrients (e.g., serum β-carotene or dietary vitamins A, C, E, selenium, zinc, and carotenoids) reported significant effect modification [65, 66]. It is possible that the use of the composite OBS that included a variety of dietary and lifestyle anti- and pro-oxidants may have obscured modifying effects of some of the more potent individual antioxidants.

The present study has a few notable strengths, including the use of a mixture exposure approach with both phthalate and emerging terephthalate metabolites, mirroring real-life and current-day exposure patterns. We further applied permutation tests which improves power compared to traditional WQSr methodology [40]. Furthermore, we assessed a potential mechanism linking phthalate and terephthalate exposure with cardiometabolic risk factors and explored possible solutions to attenuate the mixture exposure/outcome associations. The present study is not without limitations. The cross-sectional nature of NHANES prevents causal interpretation of the findings. Additionally, because phthalate metabolites, GGT, and cardiometabolic outcomes were measured concurrently, the temporal ordering required to establish mediation cannot be confirmed. We utilized GGT as a biomarker of whole body oxidative stress, but other oxidative stress biomarkers may better capture the oxidative stress-related damage (e.g., DNA and lipid-related) that phthalate exposure may impose [19–21]. However, serum GGT has been identified as an oxidative stress biomarker [37], and by excluding participants with self-reported liver disease we reduced potential confounding from overt hepatic dysfunction. Residual confounding due to subclinical liver disease remains possible. Additionally, OBS components were based on self-reported diet and lifestyle data and therefore may be subject to bias. While the use of WQSr is a strength because it allowed us to estimate the overall effects of correlated phthalate and terephthalate mixtures and identify the most influential metabolites, the method assumes linear, additive effects and does not account for interactions among mixture components. Inference for each WQS model was based on a permutation test, which provides valid p-values for WQSr [40]. Nevertheless, because we evaluated multiple cardiometabolic outcomes and conducted several sensitivity analyses, some statistically significant findings may have occurred by chance. Finally, LDL-C was calculated using the Friedewald equation rather than directly measured, which may result in some degree of measurement error.

In conclusion, we observed that exposure to a mixture of phthalate and terephthalate was associated with altered biomarkers of glucose and lipid metabolism. The terephthalate metabolite MEHHTP dominated the mixtures positively associated with the glycemic outcomes. We also observed that serum GGT mediated some of these associations, while a composite score of dietary and lifestyle antioxidants attenuated the impact of phthalate/terephthalate mixture exposure on HDL-C and TC/HDL-C ratio among females. These findings suggest that reducing exposure to phthalates/terephthalates, such as by limiting the consumption of highly processed foods and the use of phthalate-containing personal care products [67], and increasing exposure to dietary and lifestyle antioxidants may reduce the cardiometabolic impacts of these chemicals. Future prospective studies and intervention trials are required to build on our understanding of the potential impact of phthalate alternatives on cardiometabolic risk factors and explore potential solutions to counteract these effects.

## Supplementary Information

Below is the link to the electronic supplementary material.

Supplementary Material 1

## Data Availability

NHANES data are publicly available and can be downloaded from the NCHS: https://wwwn.cdc.gov/nchs/nhanes/.
