# Supplementary material for: Phthalate and Terephthalate Mixtures, Cardiometabolic Risk Factors, and the Role of Oxidative Stress: Evidence from NHANES 2015–2018
Source: Cardiovasc Toxicol. 2026 Sep 11;26(10):107. doi: 10.1007/s12012-026-10188-7 (PMC13569515; doi:10.1007/s12012-026-10188-7)
Supplement: Supplementary file 1 [file 12012_2026_10188_MOESM1_ESM.docx]

**Supplementary Material**

**Phthalate and terephthalate mixtures, cardiometabolic risk factors, and the role of oxidative stress: evidence from NHANES 2015–2018**

Mary D. Webb^a^, Sheela Sathyanarayana^b,c^, Drew B. Day^d^, Jillian C. Trabulsi^a^, Jee Won Park^e^, Melissa M. Melough^a*^

^a^Department of Health Behavior and Nutrition Sciences, University of Delaware, Newark, DE 19713, USA

^b^Center for Child Health, Behavior, and Development, Seattle Children’s Research Institute, Seattle, WA 98101, USA

^c^Department of Pediatrics, University of Washington, Seattle, WA 98195, USA

^d^Independent Resarcher, NJ, USA

^e^Department of Epidemiology, University of Delaware, Newark, DE 19713, USA

^*^Corresponding author: Address correspondence to Melissa M. Melough, Department of Health Behavior and Nutrition Sciences, University of Delaware, Newark, DE 19713, USA. Email: [melough@udel.edu](mailto:melough@udel.edu)

**Fig. S1** Flow diagram of participant eligibility from NHANES 2015–2018

**Fig. S2** Weights of metabolites from significant WQS regressions of associations between phthalate and terephthalate metabolites with glycemic outcomes

**Fig. S3** Weights of metabolites from significant WQS regressions of associations between phthalate and terephthalate metabolites with lipid outcomes

**Fig. S4** Effect estimates (and 95% CIs) from WQS regression sensitivity analyses for associations between phthalate and terephthalate mixtures and (A) fasting glucose, (B) fasting insulin, (C) HOMA-IR, and (D) HbA1c

**Fig. S5** Effect estimates (and 95% CIs) from WQS regression sensitivity analyses for associations between phthalate and terephthalate mixtures and (A) HDL-C, (B) LDL-C, (C) total cholesterol, (D) total cholesterol to HDL-C ratio, and (E) fasting triglycerides

**Fig. S6** Associations between individual urinary phthalate and terephthalate metabolites and glycemic (A) and lipid (B) outcomes

**Fig. S7** Predicted phthalate and terephthalate WQS coefficients and 95% CIs in association with lipid outcomes over values of (A-D) dietary oxidative balance score (DOBS) and (E-F) oxidative balance score plus supplements (OBS+) in interaction models

**Table S1** Scoring criteria for oxidative balance score components

**Table** **S2** Mediating effects of serum gamma-glutamyltransferase (GGT) on the associations between phthalate/terephthalate mixtures and glycemic and lipid outcomes

N = 5,155 with complete phthalate data

N = 4,195 with complete phthalate data and at least one glycemic or lipid outcome

N = 3,036 adults with complete phthalate data and at least one glycemic or lipid outcome

No outcome data (n = 960)

<20 years of age (n = 1,159)

No/incomplete phthalate data (n = 14,070)

N = 3,011 male and non-pregnant female adults with complete phthalate data and at least one outcome

Pregnant (n = 25)

**Aim 1:** N = 2,336 adults with complete phthalate data, at least one glycemic or lipid outcome, and complete covariate data (glycemic: n = 2,334; lipid: n = 2,335)

Missing data on ≥ 1 covariate (n = 675)

**Aim 2:** N = 2,224 participants eligible for Aim 1 with available GGT data and no history of liver dysfunction (glycemic: n = 2,222; lipid: n = 2,223)

**Aim 3:** N = 2,336 participants eligible for Aim 1 with complete OBS data (glycemic: n = 2,334; lipid: n = 2,335)

Liver dysfunction (or missing) (n = 112)

N = 19,225 from NHANES 2015–2018

**Fig. S1** Flow diagram of participant eligibility from NHANES 2015–2018

**Fig. S2** Weights of metabolites from significant WQS regressions of associations between phthalate and terephthalate metabolites with glycemic outcomes.

**Fig. S3** Weights of metabolites from significant WQS regressions of associations between phthalate and terephthalate metabolites with lipid outcomes.

**Fig. S4** **Effect estimates (and 95% CIs) from WQS regression sensitivity analyses for associations between phthalate and terephthalate mixtures and (A) fasting glucose, (B) fasting insulin, (C) HOMA-IR, and (D) HbA1c among all eligible NHANES 2015**–**2018 participants.** Models were adjusted for sex, age, race and ethnicity, education level, PIR, smoking status, alcohol consumption, physical activity, sedentary behavior, energy intake, diet quality, natural log-transformed urinary creatinine, menopause status, survey cycle, and glucose-lowering medication use, with additional adjustment for BMI, medical conditions (diabetes, liver condition, kidney dysfunction, hypertension), or both BMI and medical conditions. Effect estimates represent the unit or percent change (fasting insulin, HOMA-IR) in the outcome for a one-decile increase in the phthalate/terephthalate mixture.

Abbreviations: BMI, body mass index; Med. Cond., medical conditions

**Fig. S5** Effect estimates (and 95% CIs) from WQS regression sensitivity analyses for associations between phthalate and terephthalate mixtures and (A) HDL-C, (B) LDL-C, (C) total cholesterol, (D) total cholesterol to HDL-C ratio, and (E) fasting triglycerides among all eligible NHANES 2015–2018 participants. Models were adjusted for sex, age, race and ethnicity, education level, PIR, smoking status, alcohol consumption, physical activity, sedentary behavior, energy intake, diet quality, natural log-transformed urinary creatinine, menopause status, survey cycle, and lipid-lowering medication use, with additional adjustment for BMI, medical conditions (diabetes, liver condition, kidney dysfunction, hypertension, CVD), or both BMI and medical conditions. Effect estimates represent the unit or percent change (fasting triglycerides) in the outcome for a one-decile increase in the phthalate/terephthalate mixture.

Abbreviations: BMI, body mass index; Med. Cond., medical condition; TC/HDL-C Ratio, total cholesterol to HDL-C ratio

**Fig. S6** Associations (with 95% confidence intervals) between individual urinary phthalate and terephthalate metabolites and glycemic (A) and lipid (B) outcomes from multivariable linear regression models. Estimates represent the unit or percent change in each outcome per each natural log-unit increase in covariate-adjusted creatinine-standardized urinary metabolite concentration. Glycemic outcome models were adjusted for sex, age, race and ethnicity, education level, poverty-to-income ratio, smoking status, alcohol consumption, physical activity, sedentary behavior, energy intake, diet quality, natural log-transformed urinary creatinine, menopause status, survey cycle, and glucose-lowering medication use. Lipid outcome models were adjusted for the same covariates except lipid-lowering medication use was included in place of glucose-lowering medication use.

**Fig. S7** Predicted phthalate and terephthalate WQS coefficients and 95% CIs in association with lipid outcomes over values of (A-D) dietary oxidative balance score (DOBS) and (E-F) oxidative balance score plus supplements (OBS+) in interaction models. Models were adjusted for age, race and ethnicity, education level, PIR, sedentary behavior, energy intake, diet quality, natural log-transformed urinary creatinine, menopause status, survey cycle, and lipid-lowering medication use. DOBS models were also adjusted for smoking status, alcohol consumption, and physical activity. All models also included a product term between the WQS mixture coefficient and DOBS or OBS+. Plots are shown for models in which the product term was statistically significant (p < 0.05). P-values for the product term (interaction p) are shown within each plot.

Abbreviations: TC/HDL-C Ratio, total cholesterol to HDL-C ratio

**Table S1.** Scoring criteria for oxidative balance score components

| **Component** | **Scoring criteria** |
| --- | --- |
| *Dietary antioxidants* |  |
| β-carotene | Based on sex-specific tertile rankings:   - 0 points for tertile 1 - 1 point for tertile 2 - 2 points for tertile 3 |
| Calcium |  |
| Copper |  |
| Fiber |  |
| Folate |  |
| Magnesium |  |
| Niacin |  |
| EPA + DHA |  |
| Riboflavin |  |
| Selenium |  |
| Vitamin B_12_ |  |
| Vitamin B_6_ |  |
| Vitamin C |  |
| Vitamin E (as alpha-tocopherol) |  |
| Zinc |  |
| *Dietary pro-oxidants* |  |
| Iron | Based on sex-specific tertile rankings:   - 2 points for tertile 1 - 1 point for tertile 2 - 0 points for tertile 3 |
| Saturated fat |  |
| *Lifestyle antioxidants* |  |
| Physical activity | Based on sex-specific tertile rankings:   - 0 points for tertile 1 - 1 point for tertile 2 - 2 points for tertile 3 |
| *Lifestyle pro-oxidants* |  |
| Alcohol consumption | Based on self-reported alcohol intake:   - 2 points for none/never intake^a^ - 1 point for moderate intake^b^ - 0 points for above moderate intake^c^ |
| Smoking status | Based on self-reported smoking:   - 2 points for never smoker^d^ - 1 point for former smoker^e^ - 0 points for current smoker^f^ |
| Body mass index | Based on sex-specific tertile rankings:   - 2 points for tertile 1 - 1 point for tertile 2 - 0 points for tertile 3 |

^a^No alcohol consumption in last 12 months or ever; ^b^≤ 2 drinks/day for males or ≤ 1 drink/day for females; ^c^ >2 drinks/day for males or >1 drink/day for females; ^d^Have not smoked 100 cigarettes in lifetime; ^e^Smoked ≥ 100 cigarettes in lifetime but do not currently smoke cigarettes; ^f^Smoked ≥ 100 cigarettes in lifetime and currently smoke cigarettes every day or some days

**Table S2**. Mediating effects of serum gamma-glutamyltransferase (GGT) on the associations between phthalate/terephthalate mixtures and glycemic and lipid outcomes. Mediation effect estimates represent the unit or percent (fasting insulin, HOMA-IR, fasting triglycerides) change in the outcome for a change from the first to third quartile of the phthalate/terephthalate mixture exposure (i.e., WQS index).

| **Outcome** | **Direction** | **ACME (95% CI)** | **ADE (95% CI)** | **Total Effect (95% CI)** | **Estimated Percent Mediated^a^** |
| --- | --- | --- | --- | --- | --- |
| Fasting Glucose | Negative | **-0.68 (-1.40, -0.16)** | -1.38 (-4.79, 2.03) | -2.06 (-5.37, 1.33) | 32.84 |
| Fasting Glucose | Positive | -0.04 (-0.43, 0.43) | 4.77 (2.37, 7.62) | 4.73 (2.27, 7.49) | -0.85 |
| Fasting Insulin | Negative | -- | -- | -- | -- |
| Fasting Insulin | Positive | 0.11 (-1.61, 1.87) | 26.93 (18.42, 36.13) | 27.06 (18.21, 36.48) | 0.45 |
| HOMA-IR | Negative | -- | -- | -- | -- |
| HOMA-IR | Positive | 0.07 (-1.93, 2.13) | 30.10 (20.47, 40.37) | 30.19 (19.99, 40.52) | 0.26 |
| HbA1c | Negative | -- | -- | -- | -- |
| HbA1c | Positive | 0.00 (-0.00, 0.01) | 0.12 (0.07, 0.18) | 0.12 (0.07, 0.18) | 3.32 |
| HDL-C | Negative | -0.02 (-0.12, 0.07) | -2.73 (-3.70, -1.77) | -2.75 (-3.73, -1.79) | 0.82 |
| HDL-C | Positive | **0.11 (0.03, 0.23)** | -0.55 (-1.49, 0.26) | -0.44 (-1.39, 0.41) | -26.26 |
| LDL-C | Negative | -0.18 (-0.64, 0.24) | -5.57 (-8.49, -2.11) | -5.76 (-8.73, -2.25) | 3.19 |
| LDL-C | Positive | -0.15 (-0.59, 0.30) | 0.20 (-3.40, 4.10) | 0.06 (-3.52, 3.96) | -254.53 |
| TC | Negative | -0.45 (-0.95, 0.05) | -4.23 (-6.68, -1.68) | -4.69 (-7.08, -2.16) | 9.64 |
| TC | Positive | -- | -- | -- | -- |
| TC/HDL-C Ratio | Negative | **-0.03 (-0.05, -0.01)** | -0.06 (-0.15, 0.02) | -0.10 (-0.18, -0.01) | 34.82 |
| TC/HDL-C Ratio | Positive | 0.01 (-0.01, 0.03) | 0.14 (0.05, 0.23) | 0.15 (0.07, 0.24) | 7.02 |
| Fasting TG | Negative | **-4.27 (-6.47, -2.31)** | -3.69 (-9.02, 1.78) | -7.81 (-13.11, -2.62) | 53.73 |
| Fasting TG | Positive | 1.36 (-0.47, 3.26) | 5.81 (0.27, 12.11) | 7.25 (1.69, 13.57) | 19.24 |

^a^Percent mediated = (ACME/Total Effect) × 100; Boldface indicates p < 0.05 for ACME

Abbreviations: HOMA-IR, homeostatic model of insulin resistance; HDL-C, high-density lipoprotein cholesterol; LDL-C,
low-density lipoprotein cholesterol; TC, total cholesterol; TC/HDL-C ratio, total cholesterol to HDL-C ratio; TG, triglycerides
ACME, average causal mediation effect; ADE, average direct effect
